# Early Surgical Intervention following Inguinal Hernia Repair with Severe Postoperative Pain

**DOI:** 10.3389/fsurg.2017.00067

**Published:** 2017-11-30

**Authors:** Ferdinand Köckerling, Christine Schug-Pass

**Affiliations:** ^1^Department of Surgery, Center for Minimally Invasive Surgery, Academic Teaching Hospital of Charité Medical School, Vivantes Hospital, Berlin, Germany

**Keywords:** inguinal hernia, postoperative pain, chronic pain, tack fixation, re-intervention

## Abstract

**Introduction:**

Severe postoperative pain is an important risk factor for onset of chronic inguinal pain following inguinal hernia repair. All measures must be taken to eliminate postoperative pain.

**Materials and methods:**

This case report highlights the problems of severe postoperative pain following transabdominal preperitoneal patch plasty (TAPP) inguinal hernia repair and describes a systematic treatment path that may include surgical intervention.

**Results:**

Following TAPP operation for lateral inguinal hernia, this patient who had been operated on in an external hospital still experienced intense, stabbing inguinal pain on postoperative day 7 during movement, despite optimal pain treatment. Diagnostic examination did not reveal any findings of note. The surgical report documented that the surgeon had used metallic tacks for mesh fixation, i.e., at the pectineal line of the pubic bone, pubic symphysis, upper margin of the mesh, and for closure of the peritoneum. During surgical revision on postoperative day 7, eight tacks and the mesh were removed and, following further dissection, a new mesh was placed and fixed with glue. The patient’s intense stabbing pain resolved immediately after surgery.

**Conclusion:**

Since the results of late intervention for chronic inguinal pain are anything but satisfactory, early surgical intervention should be considered for patients with severe postoperative pain >3 days of suspected surgical origin.

## Introduction

In a systematic review of the risk factors of chronic pain after inguinal hernia repair, the incidence of clinically significant chronic postoperative inguinal pain (CPIP) with impact on daily activities ranged between 10 and 12% (1). Debilitating CPIP with severe impact on normal daily activities or work was reported in 0.5–6% of cases (1). Risk factors for CPIP with strong evidence include female gender, young age, high intensity of preoperative pain, high early postoperative pain intensity, history of chronic pain other than CPIP, operation for a recurrent hernia, and open repair technique (1). The most important factor in the development of chronic pain is immediate postoperative pain; all measures must be taken to eliminate postoperative pain (2). Postoperative pain after transabdominal preperitoneal patch plasty (TAPP) or total extraperitoneal patch plasty (TEP) is most severe on day 0, mainly on a level of 13–58 mm on a visual analog scale (VAS), and decreases to low levels on day 3 (3). There seems to be no difference in pain intensity and duration when TEP and TAPP are compared (3). Deep abdominal pain (i.e., groin pain/visceral pain) was dominant over superficial pain (i.e., somatic pain) and shoulder pain (i.e., referred pain) after TAPP (3). According to the new international guidelines of the HerniaSurge Group, immediate severe/excruciating postoperative pain raises the possibility of vascular or nerve injury (4). Furthermore, mesh fixation to the pubic bone leads to an increased incidence of chronic pain (4). Early reoperation is recommended to either exclude or manage these complications (4).

The following case report highlights the problems of severe postoperative pain after laparoendoscopic inguinal hernia repair and describes treatment options. The main focus here is on the identification of those patients likely to benefit from early surgical re-intervention, following laparoendoscopic inguinal hernia repair, because of the treatment-refractory postoperative pain persisting for more than 3 days after surgery.

## Case Report

This 54-year-old male patient had undergone TAPP operation at an external hospital because of a right, lateral inguinal hernia. The patient’s history documented status post-neurosurgical intervertebral disk resection secondary to intervertebral disk prolapse, L5/S1, some years previously. In this latest surgical report, the surgeon states that he had used metallic tacks during the operation for mesh fixation and for closure of the peritoneum. He had used the tacks for mesh fixation to the pectineal line of the pubic bone, pubic symphysis, and anterior abdominal wall. The metallic tacks had also been used for closure of the peritoneum.

**Figure 1:**
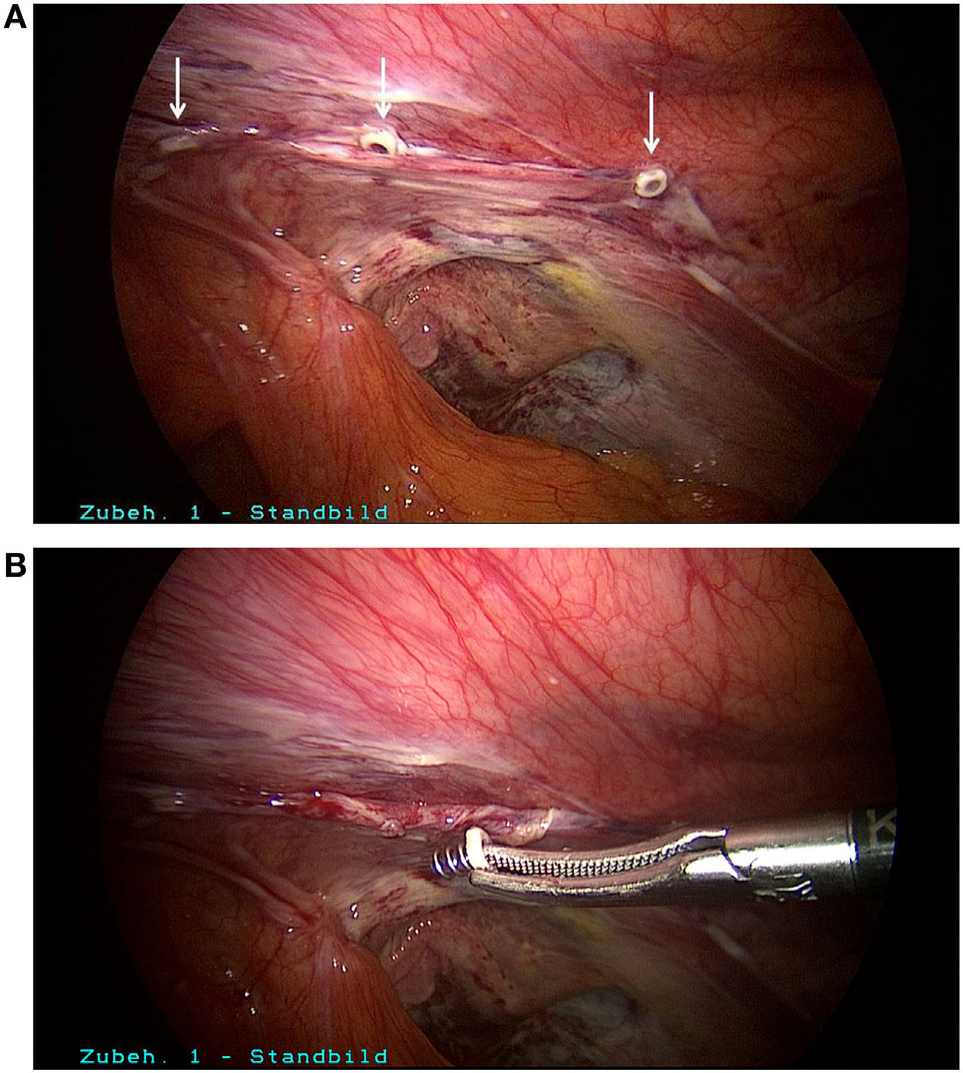
**(A)** Tacks for closure of the peritoneum. **(B)** Counterclockwise unscrewing of tacks.

**Figure 2:**
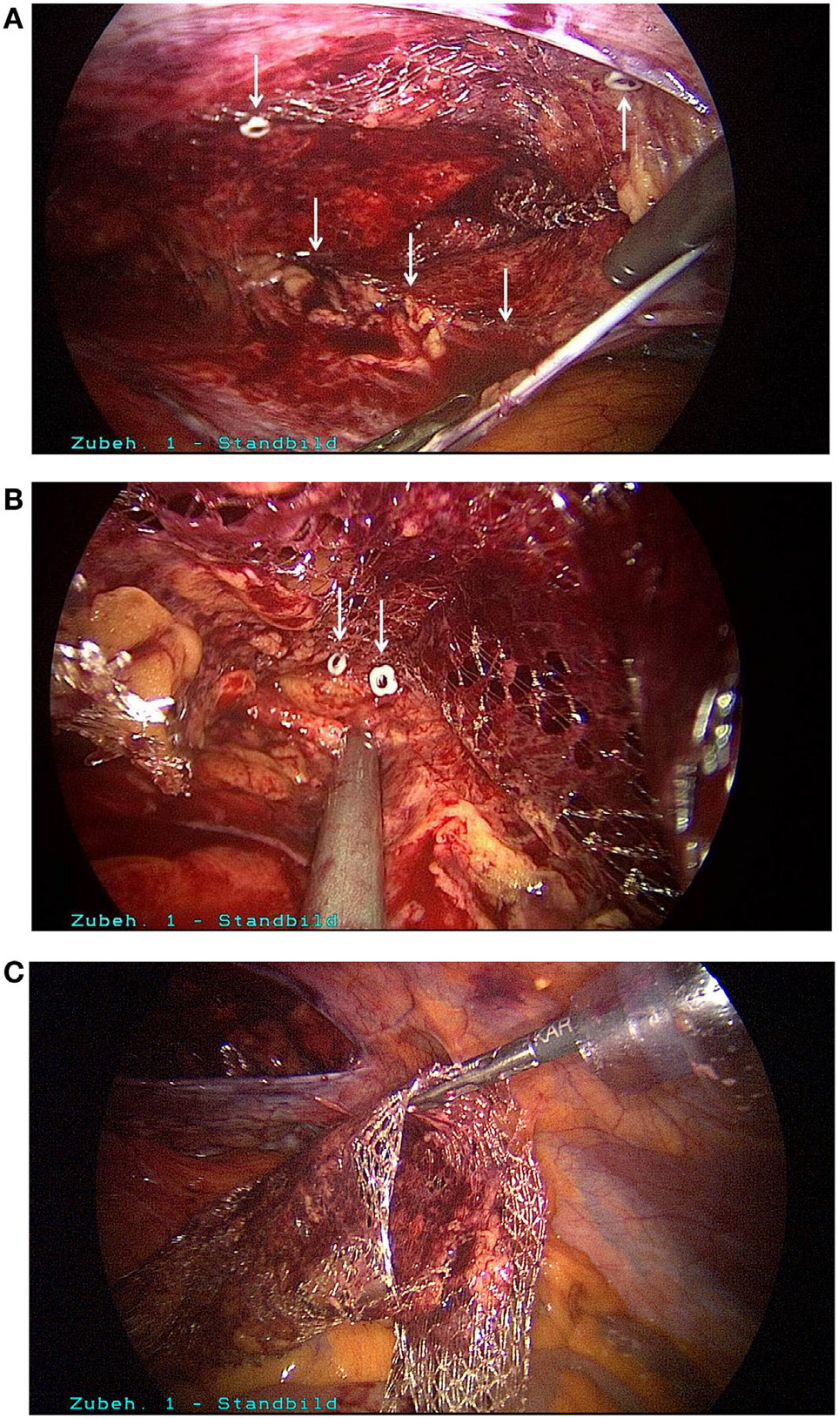
**(A)** Detection of other tacks, used for fixation of upper margin of the mesh, and of a turned-up lower mesh margin. **(B)** Search for tacks at the pectineal line of the pubic bone and symphysis. **(C)** Mesh withdrawal after removal of tacks.

**Figure 3:**
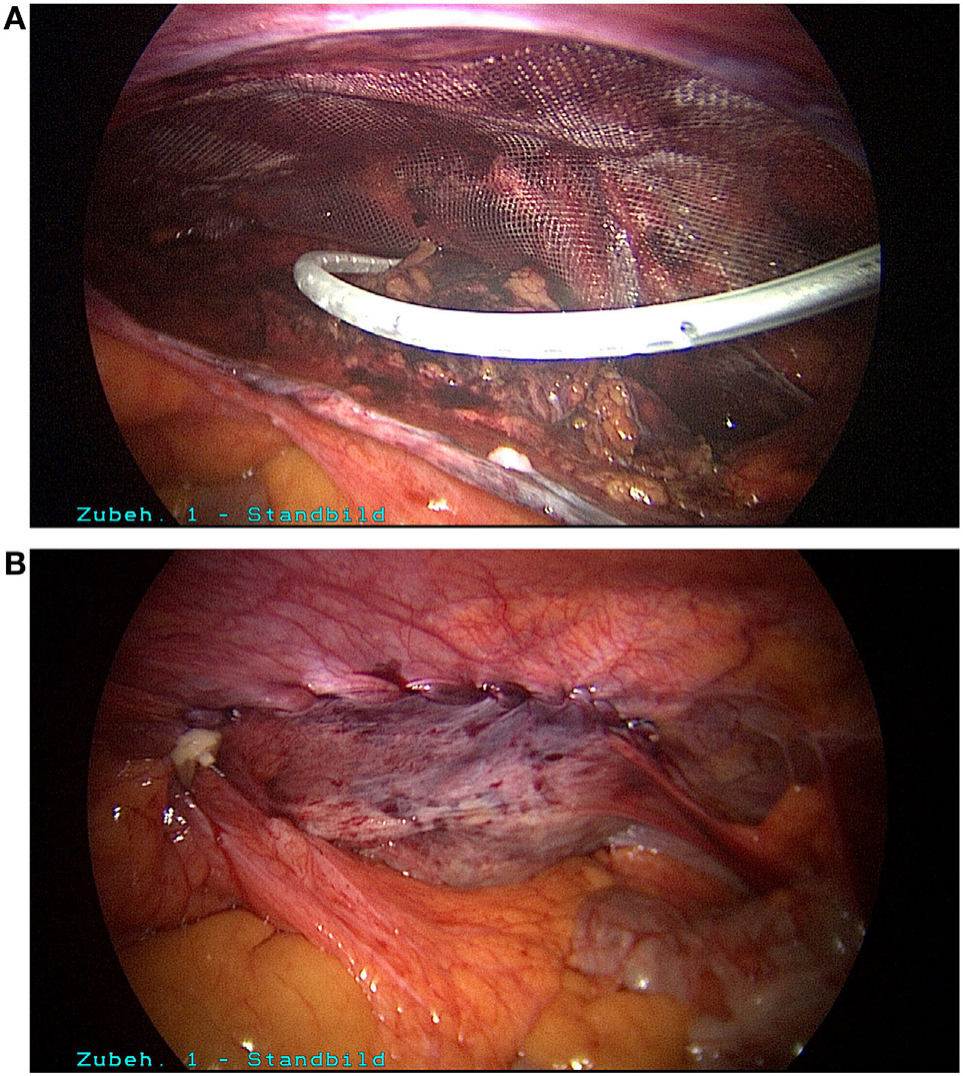
**(A)** Correct placement of new mesh (TiMesh light), fixation with fibrin glue and placement of a drain. **(B)** Closure of peritoneum with a V-lock suture.

Postoperatively, the patient experienced the most intense, stabbing pain in the groin which could not be sufficiently controlled with the maximum dose of non-steroidal antirheumatic agents prescribed by the surgeon. Since there was still no improvement on postoperative day 5, the patient presented to our hospital. We first of all ruled out the presence of a hematoma/seroma or another cause for the pain by means of ultrasound examination of the right groin. Next, morphine derivatives and gabapentin were added to the pain treatment regimen. Since there was still no improvement following optimization of pain treatment, after in-depth discussion of the situation with the patient we decided to perform surgical re-intervention in view of the fact that the surgical report documented the use of metallic tacks to the pectineal line of the pubic bone and the symphysis. That was carried out on postoperative day 7. During laparoscopic revision of the surgical area, three tacks used for closure of the peritoneum were identified (Figure 1A). These were easily gripped by the head and removed by unscrewing counterclockwise (Figure 1B). After folding down the peritoneum, other metallic tacks, used for mesh fixation at the upper margin of the mesh, were located (Figure 2A). Besides, it was revealed that here the mesh was not lying evenly on the abdominal wall; instead, its lower portion was turned-up. The reason for that was shown to be inadequate dissection in the region of the spermatic cord structures and retroperitoneum. Through further dissection, it was possible to locate and remove the tacks in the region of the pectineal line of the pubic bone and symphysis (Figure 2B). After removal of all tacks, it was then possible to withdraw the mesh, because the macroscopic appearance was not satisfying and there is no clear recommendation in the literature (Figure 2C). Next, further dissection was carried out in the critical areas, a new mesh (TiMesh light, pfm medical, Cologne) was properly inserted, fixed with fibrin glue, and a drain placed (Figure 3A). Then, the peritoneum was closed with a continuous V-lock suture (Medtronic, Meerbusch) and blocking PDS clip (Figure 3B). While releasing the CO_2_ gas *via* the drain, it was easier to check whether the re-established peritoneum was pressing the mesh properly against the abdominal wall. Postoperatively, the patient stated he no longer experienced the severe, stabbing pain in the groin. The patient became free of symptoms in the early postoperative course.

## Discussion

The case report presented here demonstrates the absolute necessity to comply with the existing guidelines for conduct for laparoendoscopic inguinal hernia surgery (4–9). All guidelines recommend that traumatic mesh fixation to bone with tacks should be avoided since that poses a higher risk for onset of chronic inguinal pain (4). There is generally no need at all for fixation for defects measuring up to 3 cm or glue fixation can be used (4–6). If traumatic fixation with tacks is needed, in particular for large medial inguinal hernias, such tack fixation should be confined to the upper margin of the mesh and not to the bone (4–6). Moreover, preference should be given to absorbable tacks. Likewise, the extent of dissection for optimal mesh placement and prevention of mesh folding should be chosen in accordance with the guidelines. Only in that way can the desired good outcome be assured for the laparoendoscopic technique.

**Figure 4:**
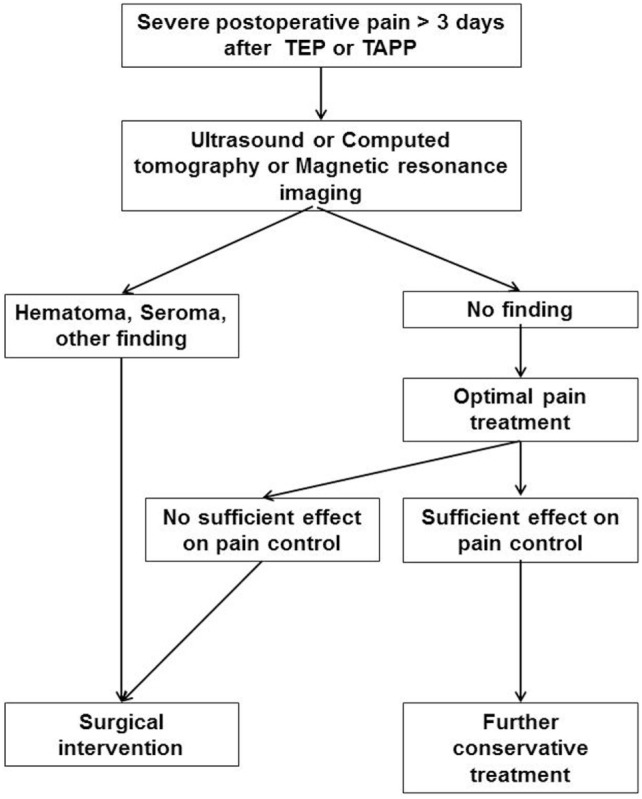
Treatment path of a patient with severe postoperative pain > 3 days after total extraperitoneal patch plasty (TEP) or transabdominal preperitoneal patch plasty (TAPP).

If a patient continues to have intense pain for more than 3 days after TEP or TAPP, and which does not respond to optimal pain treatment, the causes must be investigated. As a first step, examination with ultrasound, computed tomography, or magnetic resonance imaging should be carried out to exclude hematoma, seroma, or another morphological cause (Figure 4). If such a cause is identified, early surgical intervention must be discussed. If diagnostic exploration has not found any morphological cause for the persistent, intense postoperative pain, optimal pain treatment should be initiated in collaboration with neurologists or pain therapists. If that does not prove successful, surgical intervention must be considered even in the absence of a tangible cause. This is all the more urgent if the previous operation points to a possible explanation for development of this severe postoperative pain. Surgical intervention should then be performed up to 7 days after the primary operation. In view of the challenges encountered in treating chronic inguinal pain, with few satisfactory results, every effort must be made to prevent development of chronic inguinal pain (10). Here, the phase comprising the first seven postoperative days after inguinal hernia repair is of special significance (3). During this phase following inguinal hernia repair, the patient should as far as possible become pain free. If that is not the case technical problems linked to the preceding inguinal hernia repair cannot be ruled out. In a randomized controlled trial on day 7, 7 patients (4%) out of 196 patients who underwent TAPP reported VAS 6–10 (11). If there are any reasons to believe that the previous operation could be implicated in this persistent postoperative pain or if the technical recommendations set out in the guidelines had not been observed, surgical intervention should be contemplated and discussed with the patient. Because of the risk for onset of chronic inguinal pain and the limited prospects for successful treatment, surgeons must pay greater attention to the patient’s pain situation during the first 7 days after inguinal hernia repair. Early surgical re-intervention can perhaps prevent development of chronic inguinal pain.

In summary, this case report demonstrates that guideline recommendations for technical conduct of laparoendoscopic inguinal hernia repair must definitely be observed to prevent onset of chronic inguinal pain. If patients continue to experience treatment-refractory intense pain 7 days after inguinal hernia repair, a technical or complication-related cause cannot be excluded. If diagnostic examination identifies a specific cause, this must be eliminated. Even if no tangible cause is found, surgical intervention must nonetheless be considered to remedy potential technical mistakes.

## Ethics Statement

The patient gave his written informed consent for publication of his case.

## Author Contributions

FK: clinical case, literature review, and manuscript writing. CS-P: literature review and manuscript writing.

## Conflict of Interest Statement

The authors declare that the research was conducted in the absence of any commercial or financial relationships that could be construed as a potential conflict of interest.
